# Superstable Wet Foams and Lightweight Solid Composites from Nanocellulose and Hydrophobic Particles

**DOI:** 10.1021/acsnano.1c07084

**Published:** 2021-11-16

**Authors:** Roozbeh Abidnejad, Marco Beaumont, Blaise L. Tardy, Bruno D. Mattos, Orlando J. Rojas

**Affiliations:** † Department of Bioproducts and Biosystems, School of Chemical Engineering, Aalto University, P.O. Box 16300, FI-00076 Aalto, Finland; ‡ Department of Chemistry, Institute of Chemistry of Renewable Resources, University of Natural Resources and Life Sciences, 3430 Tulln, Austria; § Bioproducts Institute, Department of Chemical and Biological Engineering, Department of Chemistry and Department of Wood Science, University of British Columbia, 2360 East Mall, Vancouver, BC V6T 1Z4, Canada

**Keywords:** nanocellulose, interfacial interactions, particle-stabilized foams, colloidal foams, nanofibril, multiphase, stabilization

## Abstract

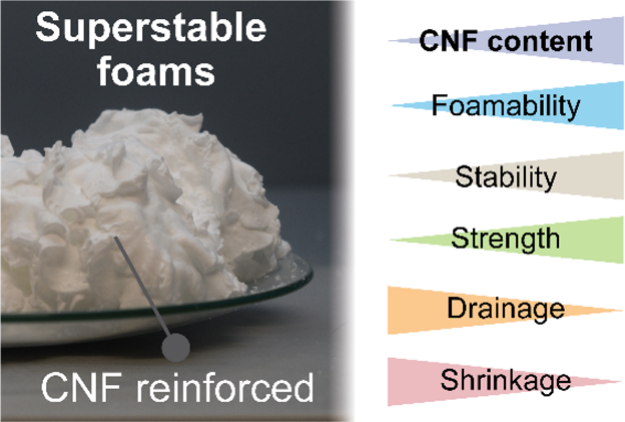

Colloids are suitable options to replace surfactants in the formation of multiphase systems while simultaneously achieving performance benefits. We introduce synergetic combination of colloids for the interfacial stabilization of complex fluids that can be converted into lightweight materials. The strong interactions between high aspect ratio and hydrophilic fibrillated cellulose (CNF) with low aspect ratio hydrophobic particles afford superstable Pickering foams. The foams were used as a scaffolding precursor of porous, solid materials. Compared to foams stabilized by the hydrophobic particles alone, the introduction of CNF significantly increased the foamability (by up to 350%) and foam lifetime. These effects are ascribed to the fibrillar network formed by CNF. The CNF solid fraction regulated the interparticle interactions in the wet foam, delaying or preventing drainage, coarsening, and bubble coalescence. Upon drying, such a complex fluid was transformed into lightweight and strong architectures, which displayed properties that depended on the surface energy of the CNF precursor. We show that CNF combined with hydrophobic particles universally forms superstable complex fluids that can be used as a processing route to synthesize strong composites and lightweight structures.

Multiphase systems consist of two or more immiscible phases that are kinetically stabilized and can be used as precursors of materials that find use in a wide range of applications.^1^ In their handling, preventing or delaying phase inversion is required for most practical uses.^2^ While some synthetic molecules (including surfactants, oligomers, and polymers) have been shown as effective stabilizers of interfaces, they are usually associated with detrimental effects, such as toxicity, allergies, and other health issues. The toxicity and allergenic concerns are important in the formulation of cosmetic and pharmaceutical products.^1,3−5^ As a key component with surface activity, surfactants might show nonspecific binding, resulting in their bioaccumulation in natural environments, including soil and aquatic streams.^6−8^ Notably, low molecular weight species are relatively ineffective in preventing Ostwald ripening or coalescence of the dispersed phase in complex fluids.^5^ Given these effects, hydrophobic colloids have been proposed to replace monomeric surfactants.^9,10^ The former display slower transport rates but adsorb more irreversibly, preventing interfacial exchange and destabilizaiton.^9^ Hence, Pickering stabilization of multiphase systems,^1,10−12^ such as emulsions and foams, endows a high resistance to coalescence and ripening.^4^ They have shown promise for the synthesis of highly stable foams, where surfactants are ineffective at preventing coalescence, given their low desorption energy (a few *k*_B_*T* for a single surfactant molecule). By comparison, under proper conditions, such as a high degree of hydrophobicity, particles can attach to the interface with characteristically higher adsorption energies, thousands of *k*_B_*T*, depending on particle size, interfacial tension, and wettability.^5^ The effect of particle hydrophobicity on its interfacial stabilization capacity is proportional to the degree of hydrophobization.^10^

Considering the effect of Pickering stabilization, it is not surprising that particles are widely reported in the formulation of foams for thermal insulation,^13^ 3D printing,^14,15^ and as sacrificial templates for the preparation of a variety of porous architectures.^5,16^ Although the benefits of Pickering stabilization are obvious in these applications, there is also a major challenge: the removal of the given fluid phase (by drying, for example) usually yields fragile networks, mainly held by van der Waals forces, leading to the collapse of the structure (isotropic shrinkage).^17^ Therefore, a practical option to compensate for the poor mechanical performance of materials obtained from foamed precursors is to add a reinforcing agent, such as a polymer, which can lead to structured interparticle interactions.^15,18^ For this reason, high aspect ratio hydrophobic colloids, such as carbon nanotubes^19,20^ and chitin nanofibers,^21^ have been introduced as a component of the complex fluids. Cellulose nanofibrils (CNF), another high aspect ratio colloid, are not as efficient in forming foams (given their hydrophilic character), unless they are chemically modified or combined with a surface-active component,^22^ which slows down drainage, coarsening, and ultimately coalescence.^22^

Cellulose nanofibrils have been shown to act synergistically with other colloids to synthesize highly robust assemblies.^23^ This is a result of the various topologies and toughening mechanisms arising from the networks that combine low aspect ratio particles and high aspect ratio fibrils. Moreover, the interconnectivity and entanglement of CNF networks induce gelation in multiphase systems at very low solid fractions,^24,25^ allowing the possibility of very light and strong materials.

Using the concepts of superstructured colloids, we propose CNF as a component of typical complex fluids, such as Pickering foams, which are stabilized by hydrophobic particles (herein, Teflon, and hydrophobized fumed silica). Unmodified, mechanically fibrillated CNF, as well as acetylated and isobutyrylated CNF, was used to enhance foamability and to dramatically improve the stability of the formed wet foams. They were used as precursors of solid foams that benefited from a very limited drainage and shrinkage during drying. Ethanol was used to regulate the surface tension of the continuous aqueous phase, thus controlling the particle–CNF colloidal behavior at the air/liquid interfaces, allowing better foaming by Pickering effects (or defoaming into homogeneous particle/CNF suspensions, Figure 1a). Highly porous foams (Figure 1b) and lightweight solids (Figure 1c) were obtained from the given wet foams by only adjusting the surface tension of the continuous phase. We systematically investigate the balance between interfibril and fibril/particle interactions and the resulting cohesion (Figure 1d). The results show that, compared to unmodified CNF, the esterified nanofibrils enhanced only slightly the foamability. Upon drying, the cohesion brought by the respective nanofibrils was independent of their surface chemistry. Overall, we unveil the role of CNF in structuring air/water interfaces, facilitating the synthesis of 3D materials. Our findings have practical implications in the formation of advanced materials from natural fibers (hydrophilic) and inorganic particles (hydrophobic), taking advantage of high aspect ratio cellulose nanofibrils that act as universal binders, following their effect on cohesive interactions.

**Figure 1:**
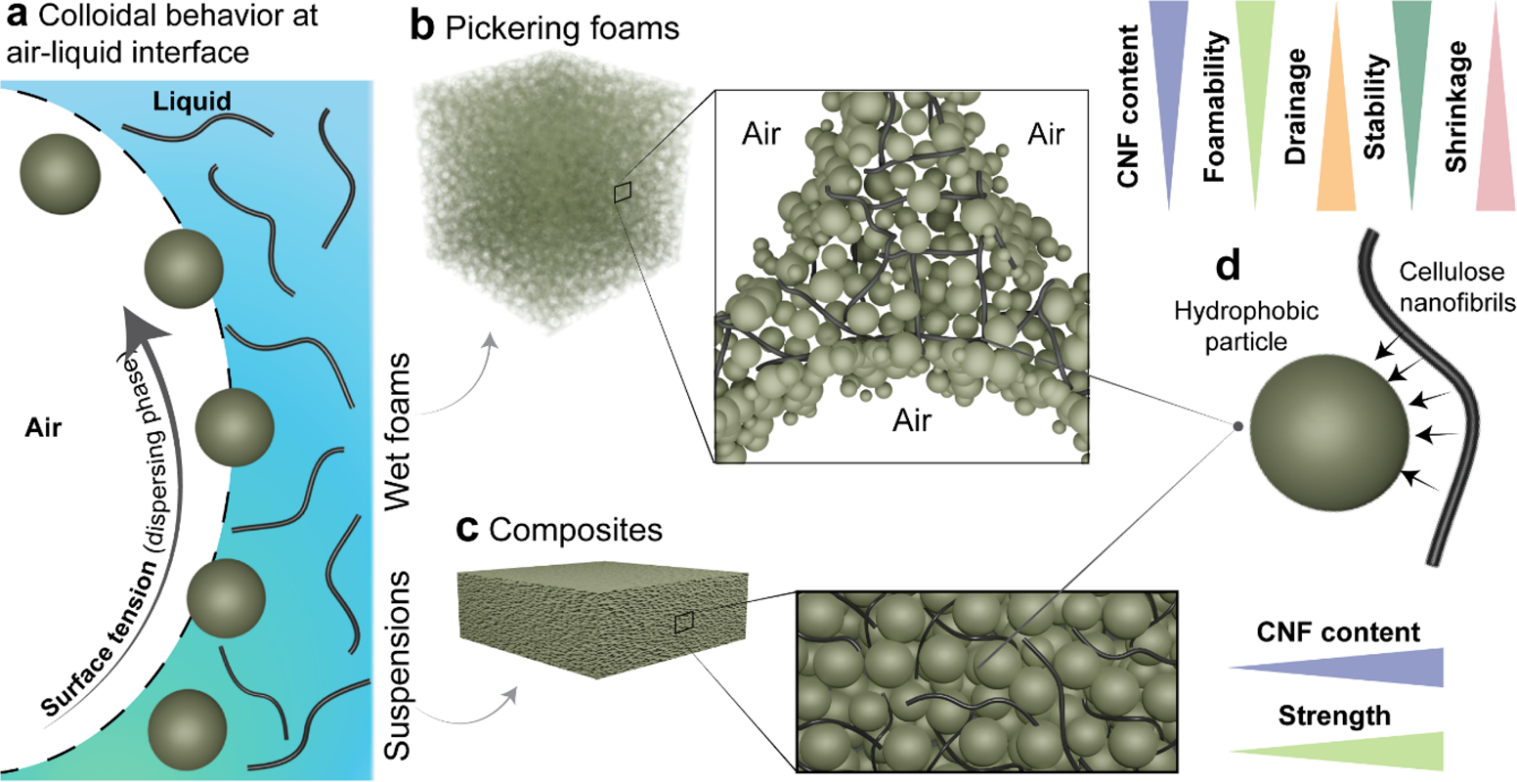
Formation of CNF–particle wet foams and lightweight composites. (a) Surface tension of an aqueous suspension containing hydrophobic particles (Teflon or hydrophobized fumed silica, shown as small spheres) can be regulated by the addition of ethanol, allowing control over the colloidal behavior at the air/liquid interface and facilitating foams and particle/nanofiber cosuspensions. (b) Wet Pickering foams are formed under a narrow range of conditions (surface tension values) of the continuous phase, whereby the foamability and stability are increased with CNF loading (conversely, drainage and shrinkage are reduced by CNF addition). (c) Robust hydrophobic particle/nanofiber constructs are obtained from their cosuspensions, formed under conditions of low surface tension (by ethanol addition), allowing homogeneous dispersion of the hydrophobic particles with a matrix formed by the cellulose nanofibrils. The constructs’ strength scales with the CNF content. (d) In the dry state, particle/CNF interactions (either in dry foams or in solid materials) are dominant and can be used to transfer cohesion in both loose and tight particle networks. CNF loading favors foamability and stability. Conversely, it reduces fluid drainage in wet foams and shrinkage upon drying.

## Results and Discussion

### Wet Foams Stabilized by Hydrophobic Particles and Cellulose Nanofibrils

On their own, CNFs perform poorly as a stabilizer of liquid foams. In fact, hydrophobization is required for CNFs to adsorb at the air/water interface, leading to bubble and foam stabilization.^26^ Herein, we used hydrophobized fumed silica to generate aqueous foams that were further stabilized with CNF (note: upon drying, the CNF also acted as a mechanical reinforcement of the system). In this discussion, we generally refer to “particles” to indicate the low aspect ratio Teflon or methylated fumed silica. Conversely, the high aspect ratio component consisted of CNFs in their unmodified or modified forms. The latter were produced by esterification, as acetylated (CNF-AA) and isobutyrylated (CNF-IBA) cellulose nanofibrils.

**Figure 2:**
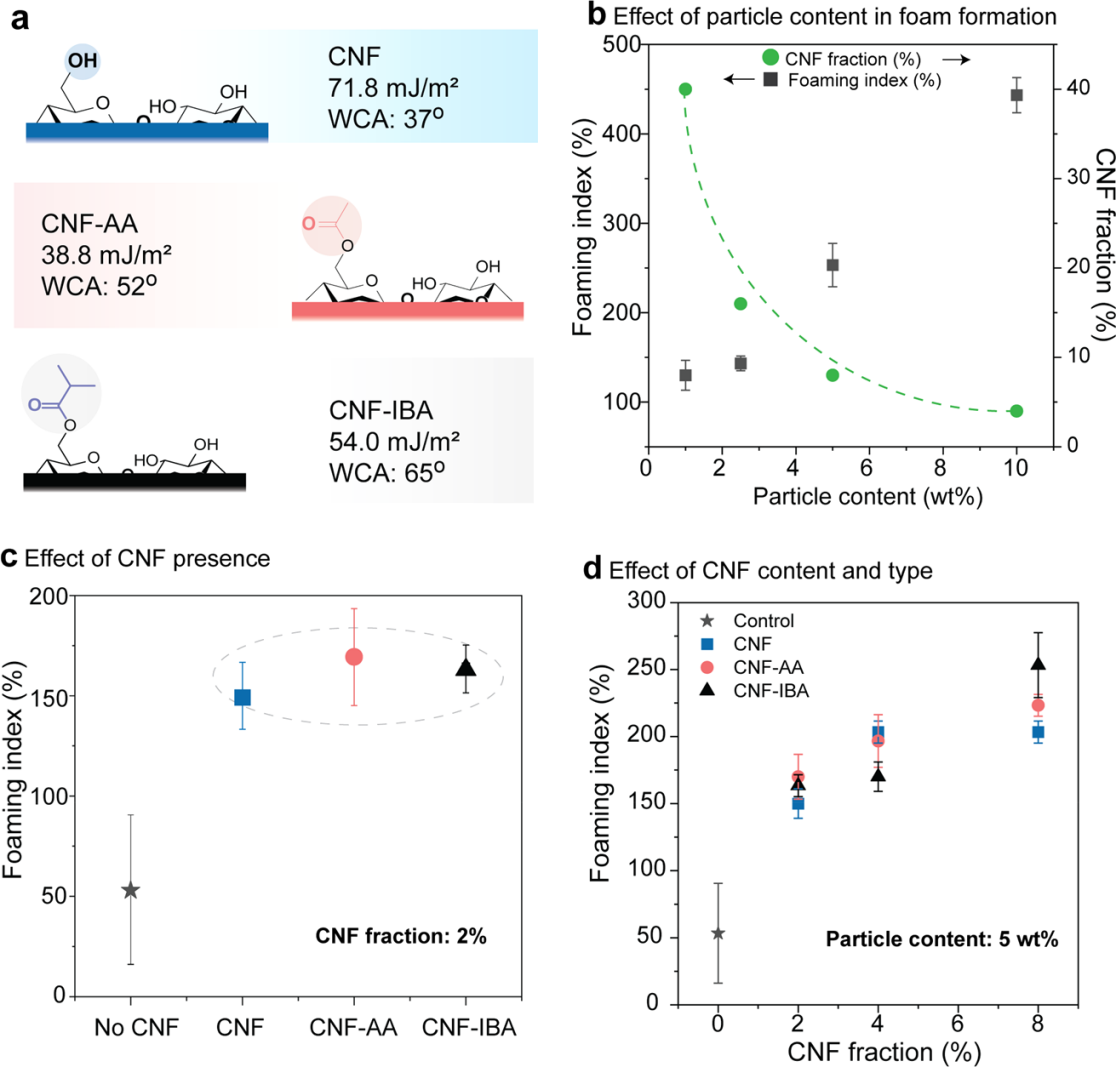
Effect of CNF type and content in foam formation. (a) Chemical structure and associated surface properties of unmodified and esterified cellulose nanofibrils: unmodified CNF (top), acetylated CNF (CNF-AA, middle), and isobutyrylated CNF (CNF-IBA, bottom). (b) Effect of the particle loading at a given CNF content on the foaming index. The variation of the CNF fraction relative to the particle content is also shown in (b). (c) Effect of CNF type on the foaming index. (d) Combined effect of CNF type and fraction on the foaming index. Note: the dashed lines in (b) and (c) are added to guide the eye and to highlight the data groups.

For foam generation, the surface tension is of primary importance as it affects particle wetting and hence the colloidal behavior at the air/liquid interface.^26^ For the precursor aqueous phase, ethanol was added at a given water-to-ethanol ratio, namely, to adjust the surface tension of the system, from 72.6 to 27.9 mN m^–1^ (Table S1).^27^ Generally, the highest foam volume was observed in Pickering foams, at the highest particle loading, when using 70:30 H_2_O/EtOH ratio (corresponding to 35.0 mN m^–1^) (Figure S1). Meanwhile, well-dispersed particle/CNF cosuspensions were formed at even higher ethanol fractions, with no signs of phase separation (hence, the system with higher ethanol fraction was used as the precursor of dry 3D constructs, as will be discussed in the respective section). Here, we first evaluate the effect of CNF type and relative content (Figure 2) on foamability and stability, noting that the CNF fraction (%) refers to the relative mass of CNF with respect to that of the hydrophobic particles.

Esterified cellulose nanofibrils (Figure S2), with hydrophilicity relatively lower than that of the unmodified CNF (Figure S3 and Figure 2a), were used to improve particle/fibril interactions as well as interfacial (air/water) stability.^23^ This was a result of the balance of surface polarity, considering the interactions between the unmodified (hydrophilic) or modified CNF with the hydrophobic particles. In wet foams, the fibrils and particles interacted, forming a loose, porous network. Therein, the presence of CNF, in general, prevented foam collapse and specifically resulted in reduced drainage, coalescence, and ripening. In later stages, upon drying, CNF also reinforced the foam against drying stresses. CNF/particle mixtures were added to the aqueous phase (70:30 H_2_O/EtOH), which easily produced foams under gentle agitation or shaking. The foam index (Figure 2b–d), that is, the relative volume of the foam compared to the initial volume of the suspension, was used as a measure of foamability and to track the foam stability over time.

The CNF fraction (%) was adjusted considering the particle (1 to 10 wt %) and CNF (0 to 0.4 wt %) content in the foams, for a maximum of 40% relative to the total mass of the final foam (Figure 2b). We limited the solid CNF fraction to ∼10% to attain a maximum cohesion (as will be discussed later) and to more effectively appraise the effect of fibril/particle interfacial interactions. The hydrophobic particle content was scaled, though not fully linearly, with the foam index. Increasing the particle content from 1 to 2.5 wt % only had a limited positive effect on the foam index; by contrast, particle additions above 2.5% increased foamability, following a linear scaling (Figure 2b). A minimum number of particles, depending on their size, was needed to cover the air bubbles during foaming; therefore, the particle content set a lower threshold for Pickering stabilization. A similar effect applies to surfactants.^22^ Furthermore, the presence of a small amount of CNF (both unmodified or modified) in the foam precursor, as small as 2%, led to a positive impact on the foaming index. First, we emphasize that both CNF and the particles developed a synergistic effect in the multiphase system; for example, they enabled a well-stabilized system. However, in the case of the esterified CNF, it partially acted as foaming agent, given their relatively lower surface energy (38.8 mN/m in the case of CNF-AA and 54.0 mN/m for CNF-IBA)^28^ compared to that of the unmodified CNF (71.8 mN/m). In fact, while unmodified CNF created strong networks, even at low volume fractions,^23,29^ it cannot stabilize air/liquid interfaces unless the surface energy was reduced (by surface modification nor by combination with surface active molecules).^22,30,31^ In our system, hydrophobic silica acted as a foaming component. At the same time, CNF endowed structuring at the foam plateau and reduced drainage. Meanwhile, CNF also increased particle jamming (given the increased apparent viscosity). As can be observed in Figure 2c, in the presence of CNF, the foam index increased from 50 to greater than 140–160%. However, the CNF concentration (in the foam precursor) should be limited to <0.4 wt % to avoid excess viscosity, which would limit the effective incorporation of air in the system. Overall, the foaming index is in the same range for all CNFs; however, esterified CNFs induced slightly better foaming. This was potentially a result of the stronger interfacial interactions with the hydrophobic particles.

The foam index increased with CNF fraction (regardless of CNF type) from 2 to 8% and at a given particle content (Figure 2d). In addition to the presence of the particles, CNF loading positively impacted the foam index of the wet Pickering foams. Moreover, at low CNF fractions, both CNF-AA and CNF-IBA cellulose nanofibrils provided a slightly higher foam index. By contrast, the foam index remained unchanged when comparing the results at 4 and 8% loading of unmodified CNF.

### Effect of Hydrophobic Particles and CNF on Pickering Foam Stability

**Figure 3:**
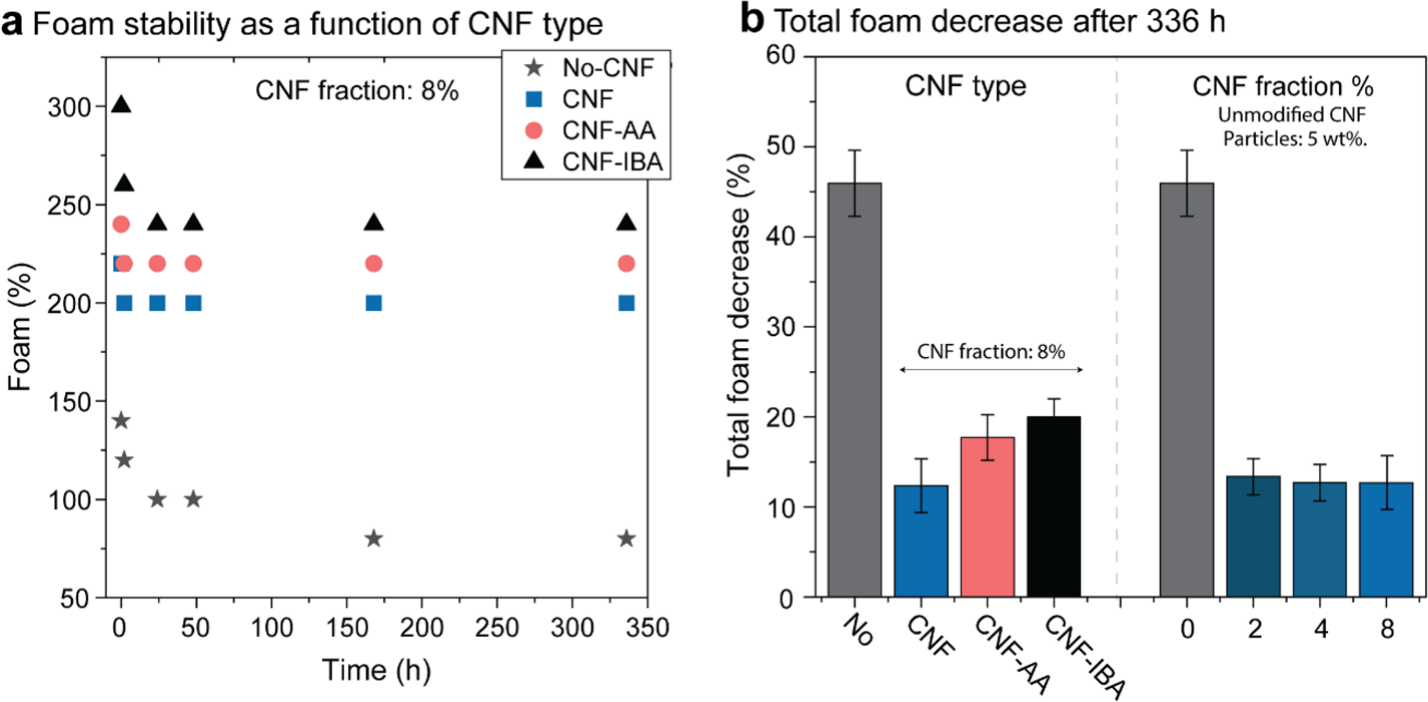
Stability of the particle/CNF Pickering foams. Effect of the (a) type of cellulose nanofibers and (b) foam volume reduction after 336 h for the different compositions and loadings (CNF type and fraction).

While the concentration of the hydrophobic particles had a dominant role on the foamability, the stability of the foam depended on the CNF type and fraction (Figure 3 and Figures S4 and S5). Moreover, CNF content positively impacted the stability of the foams because of a highly viscous phase that formed and surrounded the air bubbles *via* a quasi-continuous and entangled nanofiber network. Similar effects have been shown by polymerization of a soluble species (polyvinyl alcohol)^15,32^ around hydrophobic particles. It is reasonable to expect that further enhancement can be achieved by introducing CNF as a co-component in particle–polymer Pickering systems.^14^

The foam stability was monitored for 14 days (336 h) using the observed foam volume with respect to the initial volume (at *t* = 0, soon after the foam was formed). The most significant foam volume reduction generally took place during the first 24 h. This is a result of free water drainage, that is, before the onset of formation of an entangled CNF network, leading to gelation. Free water drainage led to shrinkage of the wet foam but, simultaneously, increased the volume fraction of particle/CNF in the foam plateau. The free water drainage concept explains the surprising high stability of foams formed in the presence of CNF, exhibiting a near constant volume for 300 h (15–20% volume reduction, at most). Foams containing CNF remained stable for over 1 year (standing at rest at room temperature and humidity), with no significant changes in volume (Figure S6). In contrast, the foams formed in the absence of CNF underwent ∼50% volume reduction after 300 h (Figure 3). Similar phenomena were observed in foams produced by a surfactant (sodium dodecyl sulfate) in the presence of CNF, which limited water drainage, given the water-holding ability of the cellulose nanofibers.^22^

A slightly lower foam stability was observed in the presence of esterified CNF, which indicates that fibril interactions are critical in developing cohesion. For a given CNF fraction, a higher particle content led to higher wet foam stability (Figure 3b); however, we emphasize that under such conditions, the CNF mass fraction of the system (2–8%) was below the value needed to achieve maximum cohesion in the dry particle/CNF network, about ∼10% solids, as will be discussed in other sections in more detail.^23^ Therefore, to understand the role of CNF in the stability of the system, we varied the CNF fraction at a given particle loading (5%) (Figure S5). CNF had a significant effect on stability, as demonstrated in Figure 3b, for the foam volume reduction after 336 h. This is a result of CNF enrichment in the nodes and plateau borders of the foam.^22^ Interestingly, and as noted earlier, increasing the CNF fraction improved the foamability but resulted in similar foam stability (Figure 3b). Therefore, it is plausible that CNF flocs form around the bubbles, even when introduced at the lowest concentration (2% dry matter content).^22,33^ However, such loading might not be sufficient to enable dispersion and attachment of the hydrophobic particles at air/liquid interfaces. Some reports indicated that hydrophobized CNF significantly increased foam stability.^31,34^ In sum, foams carrying CNF showed better stability (slower drainage) than those in the absence of CNF.

### Scaling-up Wet Foams

**Figure 4:**
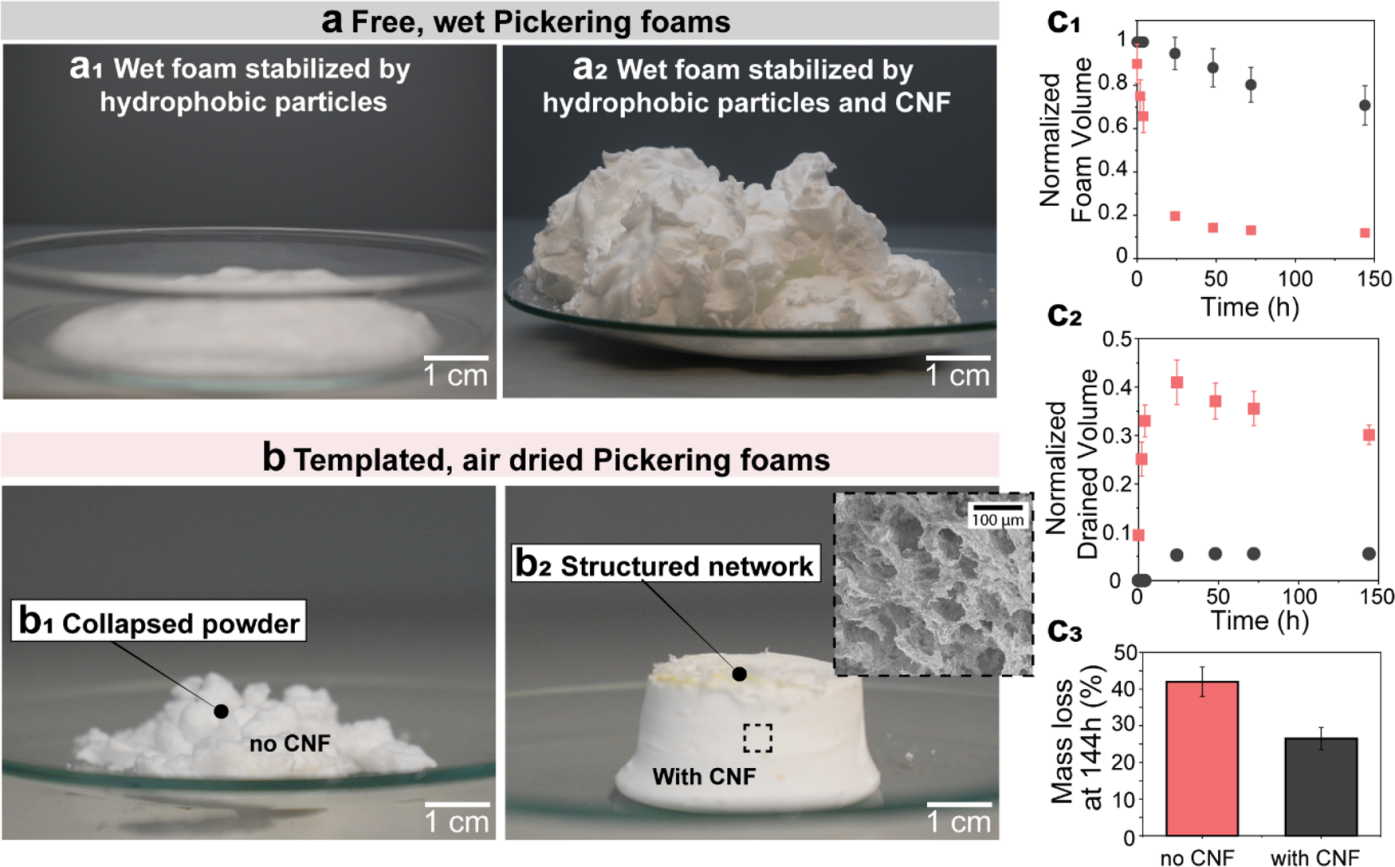
Particle-stabilized foams with or without CNF followed by consolidation in the open air. (a) Foam formed in the absence of CNF, showing no structuring (free form) (a1) and foam formed in the presence of CNF (10% dry matter) (a2), displaying an improvement in structuring. (b) Foam prepared in the absence of CNF and templated in a cylindrical glass jar (see Figure S7) that collapsed into a powder upon drying in air (b1). In the presence of CNF (10%), the templated foam maintained the initial structure, leading to a porous solid (b2 and inset). (c1,c2) Benefits of CNF addition as far as the preservation of the normalized foam volume (c1, black symbols) and reduced drainage (c2, black symbols), when compared to CNF-free foams (red symbols). (c3) Mass loss due to water evaporation is reduced in the presence of CNF.

We next demonstrate that the particle-stabilized foams can be produced at faster rates and in larger scales. The wet foams can be further used as a precursor or a template of lightweight solids *via* direct air-drying. First, we note that CNF-containing foams showed significant advantages compared with the initial and consolidated structures formed only with the hydrophobic particles (Figure 4 and supplementary Videos S1 and S2 for unmodified CNF). Compared to foams formed in the presence of CNF, those formed with the hydrophobic particles were not stable (fast drainage led to rapid bubble coalescence and foam collapse, in less than 5 min) (Figure 4a1). Upon drying, they lost the structure given the absence of interparticle cohesion (Figure 4b1). By contrast, foams retained their structure in the presence of CNF (Figure 4a2), which can be partially explained by the factors discussed previously (Figure 3). Upon drying (25% RH air), the foams co-stabilized with hydrophobic silica and CNF displayed homogeneous pores, in the range of dozens of micrometers (Figure 4b2, inset). The CNF-containing foams showed higher stability at 25% RH and 20 °C relative to those formed only by hydrophobic silica particles (Figure 4c and Figure S7). The former foams kept their structure and volume (Figure 4c1) given the absence of (or slow) drainage (Figure 4c2). The CNF-structuring and -stabilizing effect in Pickering foams was also associated with a reduced water evaporation, given the strong interactions between CNF and water. This reduces the capillary stresses generated during drying, which otherwise would lead to loose and fragile particle networks in the absence of CNF. The reduced evaporation rate was apparent in measurements of the total foam mass that was followed for 1 week observation (Figure 4c3). The complete profile of mass loss over time for foams formed with only hydrophobic particles or in the presence of CNFs further highlights the reduced evaporation rate (Figure S9), in addition to the structural superstabilization of the foams shown in Figure 4.

### Particle Consolidation for the Synthesis of Porous and Film Assemblies

As discussed previously, the 70:30 H_2_O/EtOH system induced a high foamability at high particle loadings. However, an interesting observation was that well-dispersed suspensions with the same composition of the foam precursors were obtained at a slightly increased ethanol content, ideally suited for casting in given shapes. We cast such suspensions into superhydrophobic molds and flat hydrophilic (glass) surfaces to prepare 3D architectures and films, respectively. The obtained materials are shown here as a means to understand the embedding of the hydrophobic particle within the CNF network, as well as to evaluate CNF/particle interactions and their ability to form multiscaled porous and structured materials. Ultimately, one can potentially estimate the overall cohesion of a dried foam based on its wet state and *vice versa*. In the context of other material compositions, the CNF/particle systems have been utilized in platforms that include 3D printing,^35^ insulation,^36^ catalysis,^37^ and drug delivery.^38^

**Figure 5:**
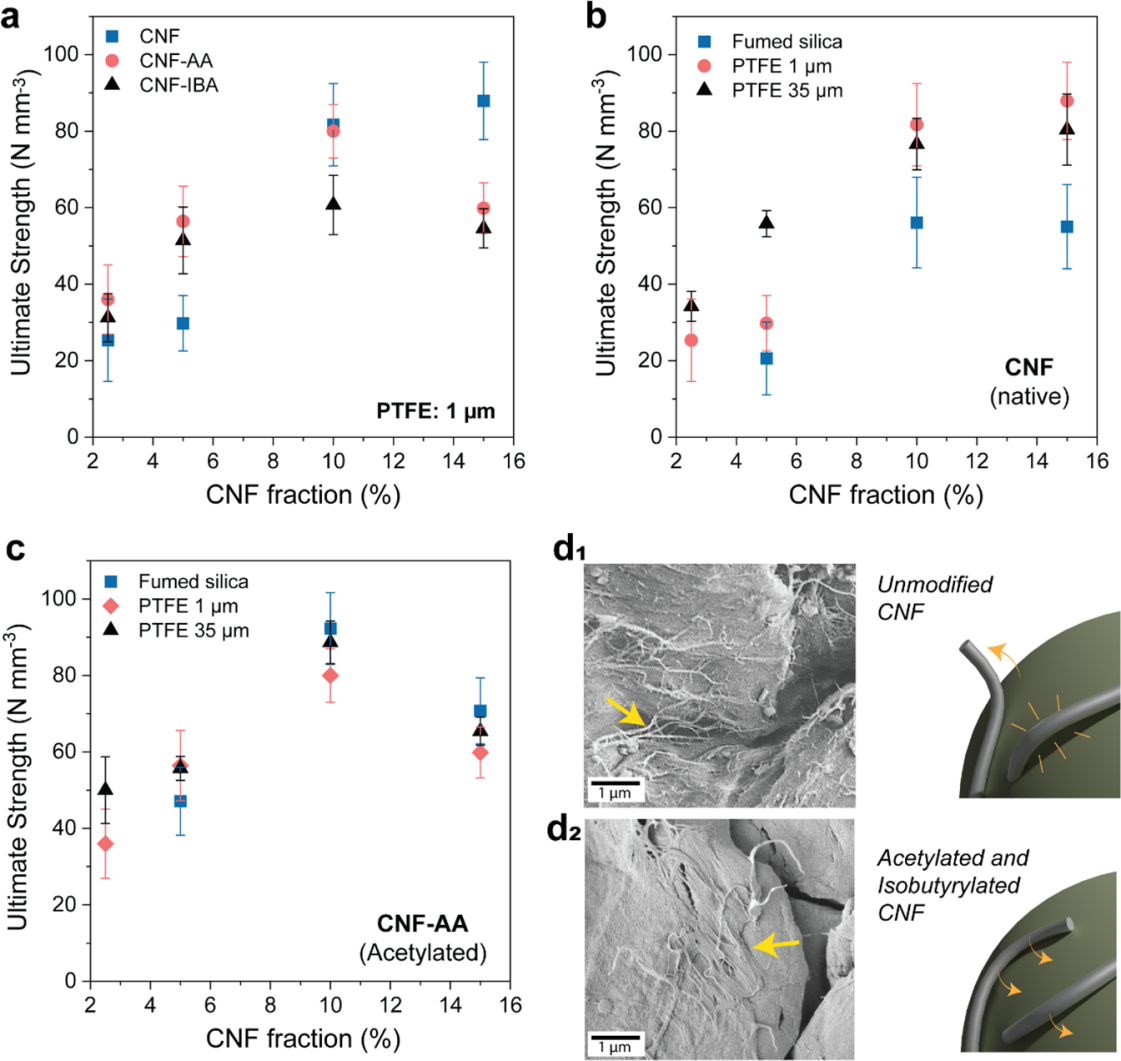
Normalized ultimate strength under uniaxial compression of particle/CNF systems obtained upon drying given precursor wet foams. (a) Effect of the CNF type on the cohesion (ultimate strength) of PTFE (polytetrafluoroethylene) particles, 1 μm. (b,c) Effect of hydrophobic particle size on the ultimate strength of the system bound by (b) unmodified CNF and (c) acetylated CNF. (d) Scanning electron microscopy images and schematic illustration showing the interactions taking place in the material, depending on CNF surface chemistry, where poorer affinity is expected for unmodified CNF (d1), whereas suitable adhesion is expected for acetylated CNF (d2). The sample compositions are as follows: PTFE 35 μm bound by 15% of unmodified CNF (d1) and acetylated CNF (d2).

Spherical particle/CNF assemblies (CNF fraction from 2.5 to 15%) were prepared by evaporation-induced self-assembly.^23^ We used various conditions to understand the effects of the interactions between the hydrophobic particles and CNF of varying surface energy and wettability (Figure 2a). We further compared the effect of CNF type and fraction as well as particle type on the cohesion of the dry materials. For such a purpose, we used uniaxial compression tests (Figure 5), with the ultimate compressive strength obtained from the force–strain curves, as shown in Figure 5a–c. We normalized the ultimate force by the volume of the spherical supraparticle (*i.e.*, N mm^–3^) to enable comparison among the prepared materials without considering impact of small macroscaled size differences.^23^ Multiparticle composites do not possess a continuous phase, and their compressive response is neither elastic nor plastic. Therefore, for instance, the Hertz model^39^ (and others) cannot be used to obtain stress values. Therefore, we prepared cylindrical foams by templating with similar compositions (hydrophobic silica and CNF fraction at 10–20%) to provide comparison points. At a CNF fraction of 10%, a compression strength up to 400 kPa at a strain of 35% was recorded (Figure S10), corresponding to ∼60 N mm^–3^ for the same composition when considering supraparticles.

Overall, the ultimate compression strength of the porous solids increased with CNF loading, from 30 to greater than 80 N mm^–3^ at CNF fractions from 2.5 to 10% (Figure 5a). No further gains were observed at higher CNF loadings. However, there were significant differences in the observed trends, depending on CNF type (unmodified and partially hydrophobized CNF), which is likely related to the effect of CNF/CNF and CNF/particle interactions. The ultimate strength of the constructs increased with CNF addition, up to 10% and for all CNF types. However, at 15% fraction, the materials produced from unmodified CNF remained largely unchanged. By contrast, the acetylated and isobutyrylated CNF led to weaker materials (Figure 5a–c).

Four types of noncovalent interactions can be reasonably assumed to take place in the formation of the solid foams: (1) weak particle/particle interactions, (2) fibril/particle interactions; (3) fibril/fibril interactions, and (4) particle/fibril interactions at the air–water interface. The balance between fibril/particle and fibril/fibril interactions dominates the cohesive strength of the consolidated constructs. Particularly, fibril/fibril hydrogen bonding interactions are expected to have a prominent role in the strength of nanocellulosic networks, in addition to their degree of entanglement.^23^ In general, particle/particle interactions, induced by long-ranged polarization effects and short-ranged van der Waals forces,^40^ dominate in the absence (or at very low loading) of CNF. With the addition of CNF, fibril/particle interactions become significant. At low CNF fraction (2.5–5%), where fibril/particle are more relevant, the modified CNF introduced higher cohesion, leading to strong particle–CNF networks, which suggests a more robust interaction in such cases (Figure 5a). At higher CNF fractions (>10%), under the dominant effects of fibril/fibril interactions, the solids prepared with unmodified CNF led to mechanically strong systems. Based on the results, it can be speculated that (1) favorable interfacial interactions and cohesion develops in constructs built from hydrophobic particles at low CNF fractions and (2) unmodified CNF binds hydrophobic particles if CNF loading exceeds 10%. When investigating the effect of particle size on CNF interactions, the smaller particles (hydrophobic fumed silica) led to slightly weaker materials bound with unmodified CNF (Figure 5b). CNFs, although very flexible building blocks, cannot create continuous networks around the very small particles^23^ such as hydrophobic fumed silica (∼7–20 nm), which leads to particle aggregates across the network that induce fracture-initiating spots, thus leading to cohesion development in the construct primarily dependent on fibril–fibril hydrogen bonding interactions. In contrast, the particles of different sizes produced materials with similar strength when bound with partially hydrophobic CNF (Figure 5c).

The effect of CNF’s surface energy on the interactions with the hydrophobic particles became apparent from image analyses (Figure 5d and Figure S8): the esterified CNF (Figure 5d2) produced a denser system with hydrophobic particles (Figure 5d1). The esterified fibrillar networks were well adhered to the particles (Figure 5d2). On the other hand, the unmodified CNF tended to peel off from the surface of the particles (Figure 5d1). Overall, regardless of the interaction type, it was clearly shown that the high aspect ratio CNF effectively binds the low aspect ratio hydrophobic particles.

**Figure 6:**
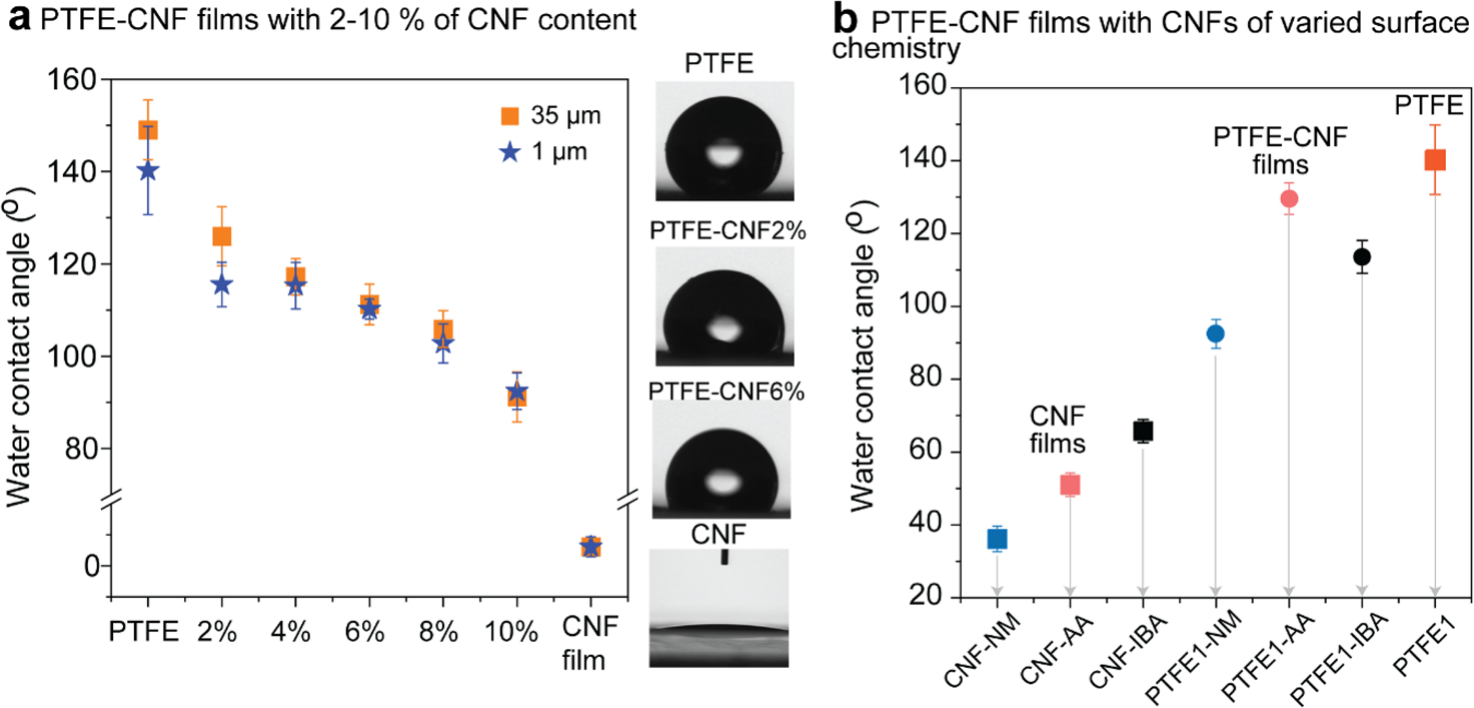
Water contact angle of CNF/PTFE composite films prepared from unmodified CNF and modified CNF (CNF-AA and CNF-IBA). (a) Effect of the CNF fraction and PTFE particle size on the WCA of the composite. (b) Effect of CNF surface chemistry on the WCA of the obtained particle/CNF films.

We prepared films from hydrophobic particles/CNF precursors to investigate, based on changes in wettability, the consistent distribution of the particles across the fibrillar hydrophilic network. As a reminder, the water contact angle (WCA) of the different CNF types used in the construct was between 37 (CNF) and 65° (CNF-IBA). Meanwhile, the WCA of polytetrafluoroethylene (PTFE) particles is >150°. Hence, an intermediate WCA value is expected when the two components are well mixed. In fact, this is the case for composite films (Figure 6): the hydrophobic particles were evenly distributed in a CNF fibrillar matrix, and the wettability scaled with the concentration of hydrophobic particles (Figure 6a). The combination of PTFE particles with CNF (all types) resulted in films of WCA ∼ 100°, which is an intermediate value compared to those of the CNF films (40–60°) and the pure PTFE particle pellets (>150°). However, the combination of PTFE particles with the esterified CNF increased the WCA (up to 130°), given the lower hydrophilicity of the respective modified CNF (Figure 6b).

We have demonstrated that CNF enables an increased foam stability that can maintain its integrity upon drying. The combination of cellulose nanofibrils and hydrophobic particles form composites with highly structured networks. This combination can be used to synthesize robust materials upon consolidation. By tuning the interfacial interactions, constructs of given morphologies can be formed, ranging from very porous foams to dense films. We highlight the prospects of transferring the cohesion of CNF networks to porous and dried particle/CNF composites, which presumably can be applied to other systems comprising at least one hydrophobic component. Based on our results, the combination of CNF (wettable, high aspect ratio) and hydrophobic particles of low aspect ratio leads to stable Pickering foams, where CNF acts as both a hydrating and a networking agent. Depending on the formulation, the wettability of the resulting dried material can be tuned from superhydrophilic to nearly superhydrophobic. Our findings are expected to have a significant impact on the design of foams (for food, cosmetics, and other applications), where stability is required. Moreover, structurally resilient dried foams can be expected with added functionalities, including insulating and nonwetting yet breathable coatings, among others. Of significance, the effect of CNF in enhancing the properties of foams, and the materials consolidated thereafter, will significantly facilitate processing, for instance, into 3D-printable inks or as building elements.

## Conclusions

Hydrophobic particles were used as foaming components toward foams that were stabilized by high aspect ratio fibrillated cellulose. In such systems, the highly hydrated CNF networks act as a mechanical lock given the effects of fibrillar crowding that reduces water drainage and leads to superstable wet foams. Upon drying, cohesive architectures are easily formed under strong cohesive forces, initially present at the foam’s plateau, which translate into strongly entangled particle/fibril architectures that retain the shape of the initial precursor system. The tight particle/fibril networks that develop by evaporation-induced self-assembly explain the high mechanical integrity and cohesion of the colloidal constructs, where fibril/fibril and fibril/particle interactions play leading roles, depending on the CNF’s surface chemistry.

## Materials and Methods

### Materials

Hydrophobic fumed SiO_2_ (silica) nanoparticles (Aerosil-R812S) with specific surface areas ranging from 195 to 245 m^2^/g were provided by Evonik Industries (CAS number 68909-20-6). The silica particles were modified with hexamethyldisilazane to make them hydrophobic. Polytetrafluoroethylene particles (1 and 35 μm, CAS number 9002-84-0) were purchased from Sigma-Aldrich. Absolute ethanol (Etax Aa, CAS number 64-17-5) was provided by Altia Company. Deionized water was used in all of the experiments.

Cellulose nanofibrils were produced from never-dried TCF-bleached sulfite dissolving-grade beech pulp (50% solid content).^41^ Three batches of disintegrated fibers (pulp) were prepared, two of which were subjected to regioselective esterification of the primary hydroxyl group (C6-OH)^42^ by *in situ* reaction with *N*-acetylimidazole and *N-*isobutyryl imidazole, yielding hydrophobic celluloses bearing acetyl (1 mmol g^–1^) and isobutyryl (0.6 mmol g^–1^) groups, respectively.^28,43^ The esterification reaction of the cellulose fibers (5 g, 31 mmol) was performed at room temperature for 24 h in the presence of 31 mL of 1 M carboxylic acid anhydride (either acetic anhydride or isobutyric anhydride) solution in acetate and 21.5 mL of 3 mL imidazole solution in acetone, according to our published procedure.^42^ The reaction was quenched by the addition of 1 mol L^–1^ sodium bicarbonate. Finally, esterified fiber suspensions were purified by dialysis (50 kDa cutoff) against DI water for 1 week (water was exchanged each day). Next, all three fiber suspensions at 1 wt % were homogenized (six passes, 200 and 100 μm chambers at 2000 bar) in a microfluidizer (Microfluidics M110P, Microfluidics Int. Co., Newton, MA), yielding CNF dispersions. Herein, the CNF samples are referred to as unmodified (CNF), acetylated (CNF-AA), and isobutyrylated (CNF-IBA) cellulose nanofibrils. The effect of fiber reaction on the chemical changes that took place on the different samples was confirmed through Fourier transform infrared and contact angle measurements of the dried materials (Figures S2 and S3, respectively)

### Methods

#### Preparation and Characterization of Pickering Foams

Wet foams were prepared with hydrophobic fumed silica particles (1 to 10 wt % of the total mass of the foam precursor) used as a foaming agent. CNF of the given type (CNF, CNF-AA, CNF-IBA) was used as a cohesion inducer and added at the respective concentrations (0.1 to 0.4 wt % of the total mass of the foam precursor). Herein, the CNF fraction (%) refers to the CNF content with respect to the particle loading in the initial foam precursor suspension, reported as % values. Water and ethanol (used to adjust the surface tension of the aqueous phase) were used in the preparation of the foams. For this purpose, a typical experiment started with the addition of fumed silica into a graduated tube, followed by the addition of ethanol to adjust the wettability. After sufficient mixing, a CNF suspension was added to the mixture. Finally, the solid concentration was adjusted with water. Each tube was then sealed, and foaming was induced by placing the system on an IKA MS2 minishaker and applying the maximum rotation speed for 60 s. Following foam formation, the tubes were handled gently and kept still for given times. The foam volume was recorded^44^ after holding times of 0, 2, 4, 24, 48, 168, and 336 h. Finally, the formulation that led to the maximum foamability (foam index) was used in experiments with larger volumes (scaling up efforts), which used a kitchen-grade creamer (Amazy-Sahnespender). The resulting foam was freeze-dried and imaged (scanning electron microscopy) following the same protocol described before.

#### Preparation of Particle/Fiber Constructs

Particle/fiber constructs were prepared with CNF (CNF, CNF-AA, CNF-IBA), used as cohesion inducer, and the particles, namely, PTFE (35 and 1 μm) and hydrophobic fumed silica. Ethanol was used to increase the wettability of the hydrophobic particles by the CNF suspensions. Water/ethanol suspensions were prepared by addition of hydrophobic particles and CNF at given composition (%), homogenized by vortexing, and consolidated by evaporation-induced self-assembly on a superhydrophobic substrate, which yielded spherical beads.^23^ Films were obtained from the same suspensions but by casting on a flat hydrophilic substrate (glass slide).

#### Characterization of the Particle/Fiber Constructs

The morphology of the constructs was investigated by field emission scanning electron microscopy (Zeiss Sigma VP, Germany) using an acceleration voltage of 1.5 kV. Prior to image capturing, samples were coated with a 4 nm gold/palladium layer with a Leica EM ACE600 high vacuum sputter coater. The mechanical strength of the spherical beads was evaluated by axial compression using a TA.XTplusC texture analysis. The measurements were taken at a compression rate of 0.10 mm/s. Finally, the water wettability of particle/fiber films was measured using a Theta Flex optical tensiometer. All measurements were done at least in three replicates.

## Supporting Information Available

Video S1 (MP4)
Video S2 (MP4)
Surface tension of ethanol–water mixtures; foam volume by ethanol ratio; FTIR and water contact angle of CNFs; additional data on foam formation and stability; photographs of foams during stability test and after 1 year of storage; additional scanning electron microscopy images of CNF–particle assemblies; mass loss of foams exposed to air; and compression curves for templated cylindrical foams (PDF)
