# Supplementary material for: Superstable Wet Foams and Lightweight Solid Composites from Nanocellulose and Hydrophobic Particles
Source: ACS Nano. 2021 Nov 16;15(12):19712–21. doi: 10.1021/acsnano.1c07084 (PMC8717629; doi:10.1021/acsnano.1c07084)
Supplement: Supplementary file 3 — nn1c07084_si_003.pdf [file nn1c07084_si_003.pdf]

Supporting Information

Superstable Wet Foams and Lightweight Solid Composites from Nanocellulose and Hydrophobic Particles

Roozbeh Abidnejad,[a] Marco Beaumont,[a,b] Blaise L. Tardy,[a,*] Bruno D. Mattos,[a,*] and Orlando J. Rojas[a,c,*]

[a]*Department of Bioproducts and Biosystems, School of Chemical Engineering, Aalto University, P. O. Box 16300, FI-00076 AALTO, Finland.*
[b]*Department of Chemistry, Institute of Chemistry of Renewable Resources, University of Natural Resources and Life Sciences, Vienna, A-3430 Tulln, Austria.*
[c]*Bioproducts Institute, Department of Chemical and Biological Engineering, Department of Chemistry and Department of Wood Science, University of British Columbia, 2360 East Mall, Vancouver, BC V6T 1Z4, Canada.*
E-mail for correspondence: bruno.mattos@aalto.fi (B.D.M), blaise.tardy@aalto.fi (B.L.T), and orlando.rojas@aalto.fi, orlando.rojas@ubc.ca (O.J.R.).

This SI file contains ten figures and one Table.

Table S1. Surface tension of DI-water/ethanol (EtOH) mixtures [25]

System	γ (mN/m)
DI-water 100 (%)	72.6
DI-water+EtOH 95/5 (%)	58.7
DI-water+EtOH 90/10 (%)	52.0
DI-water+EtOH 85/15 (%)	46.0
DI-water+EtOH 80/20 (%)	42.9
DI-water+EtOH 75/25 (%)	40.0
DI-water+EtOH 70/30 (%)	35.0
DI-water+EtOH 60/40 (%)	31.1
DI-Water+EtOH 50/50 (%)	29.2

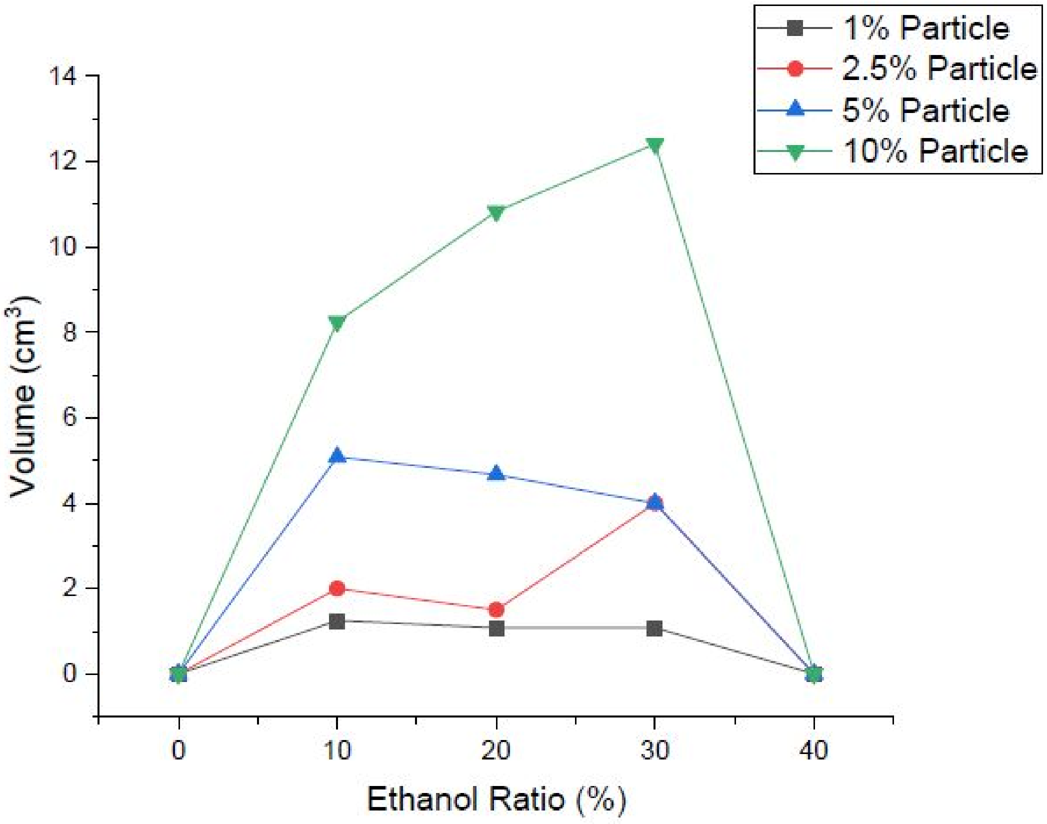

Figure S1. Effect of the ethanol ratio in suspension behavior upon low-energy agitation. The increased foam volume indicates better foamability.

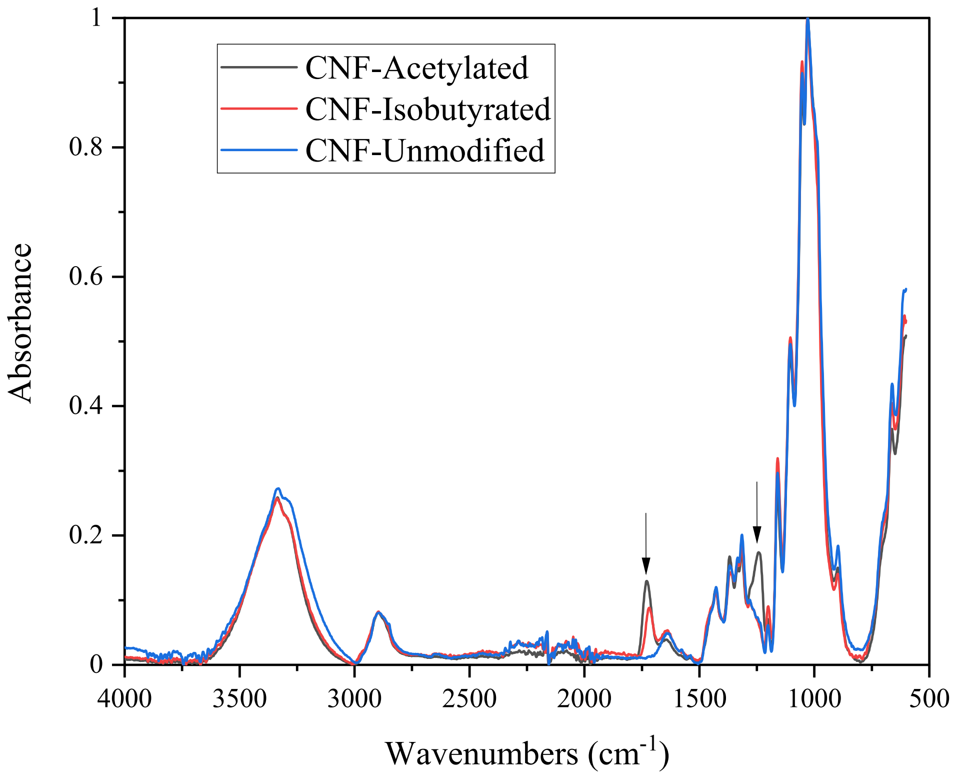

Figure S2. FTIR spectra of unmodified CNF, Acetylated CNF, and isobutyrylated CNF.

The results are shown in the Fourier Transform Infrared spectroscopy (FTIR) spectra for all CNFs – unmodified and modified – evidenced the successful esterification of the CNF samples. The appearance of a band in the range between 1718 cm^{-1} and 1729 cm^{-1}, which represents C=O stretching of the carbonyl group in the ester bond, confirms the substitution of the hydroxyl group. Also, the peak that appeared at 1241 cm^{-1} represents antisymmetric C–O stretching vibration from the grafted acetyl groups.

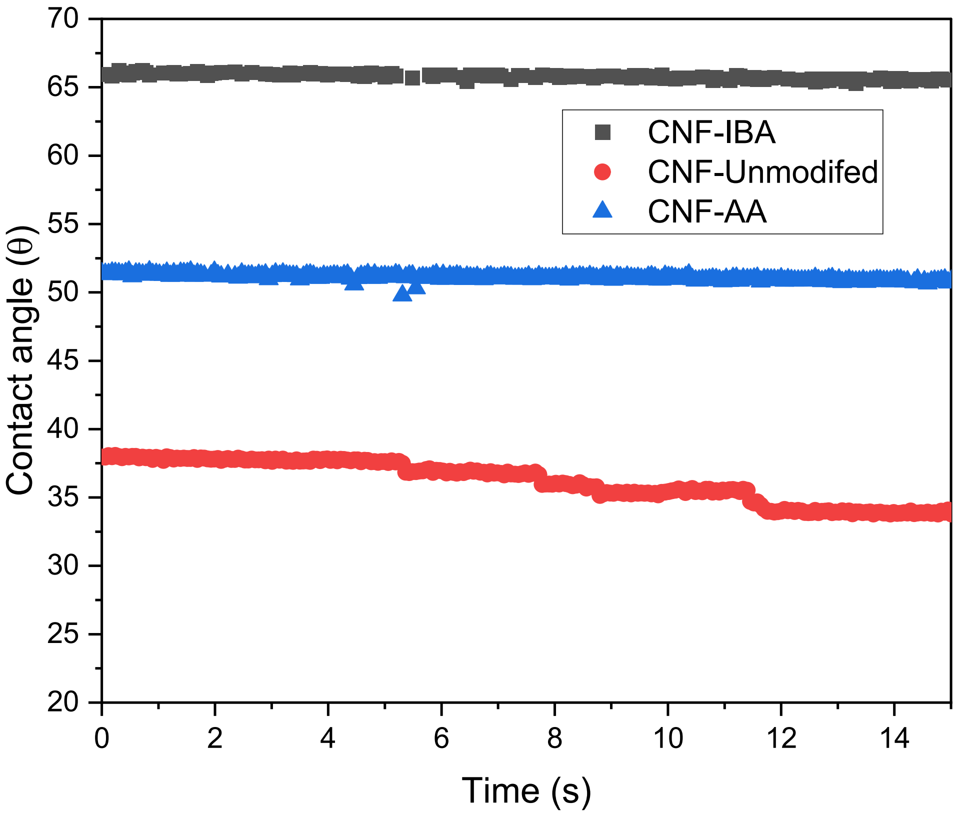

Figure S3. The water contact angle of unmodified CNF (unmodified), acetylated CNF (CNF-AA), and isobutyrylated CNF (CNF-IBA).

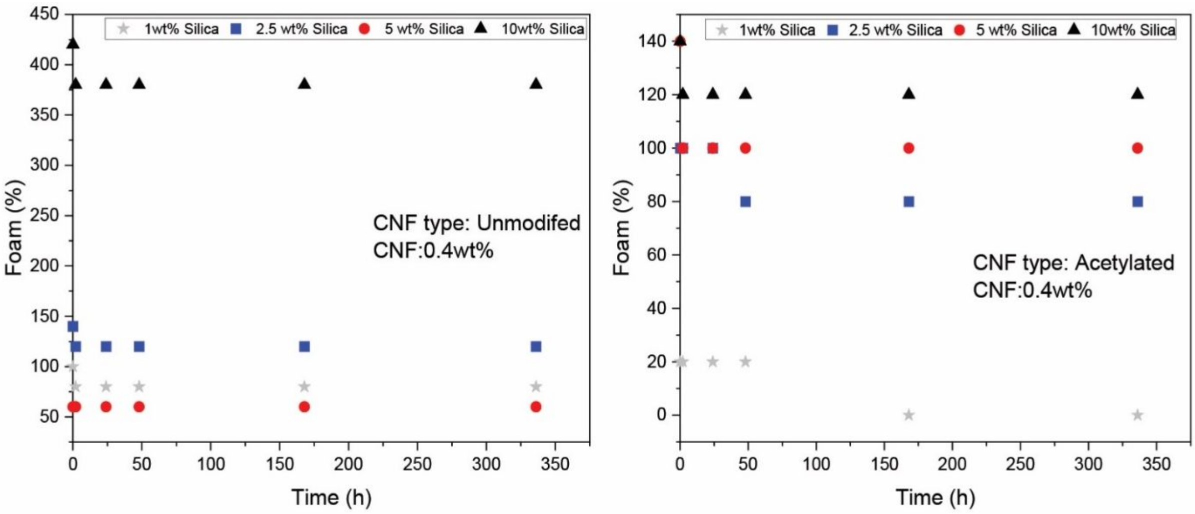

Figure S4. Stability of the particle/CNF Pickering foams prepared with unmodified (left) and acetylated CNF (right). Effect of fumed silica particle content.

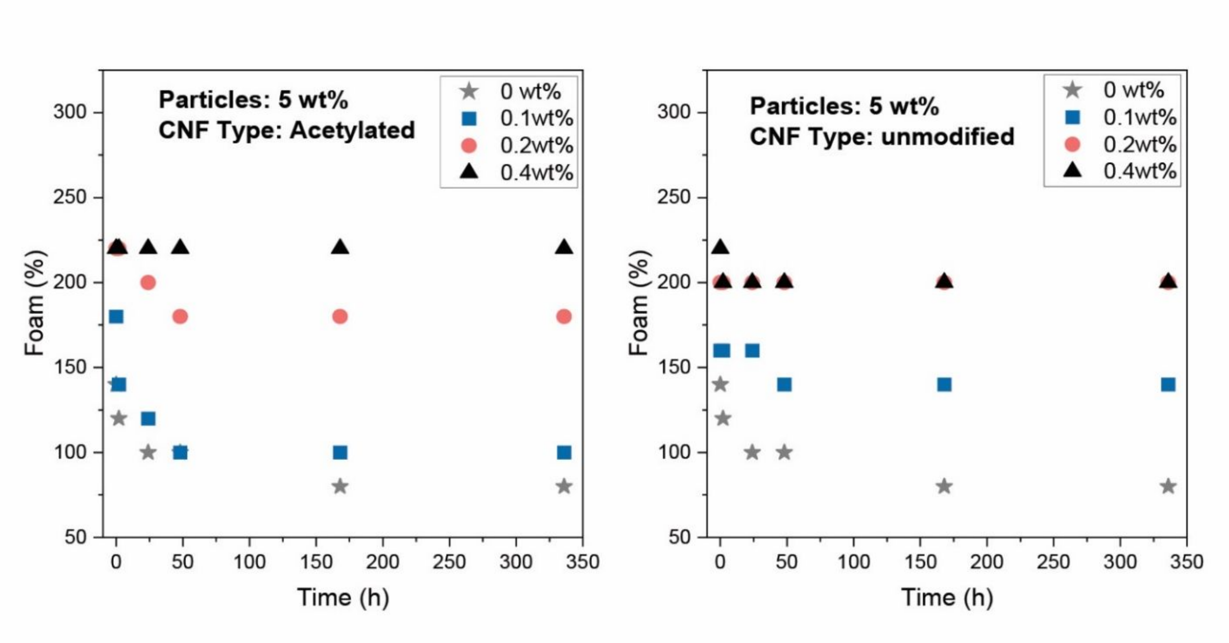

Figure S5. Stability of the particle/CNF Pickering foams prepared with unmodified (left) and acetylated CNF (right). Effect of CNF content.

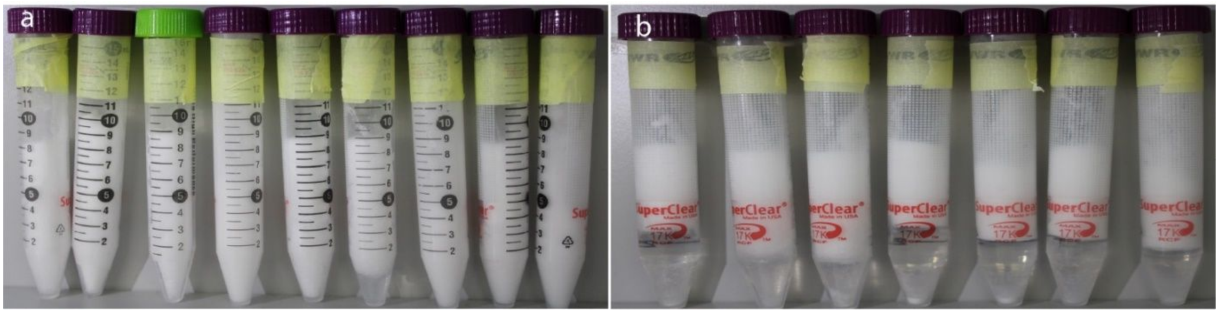

Figure S6. Photographs of the hydrophobic particle/CNFs Pickering foams at the time of production (a) and after one year of storage (b).

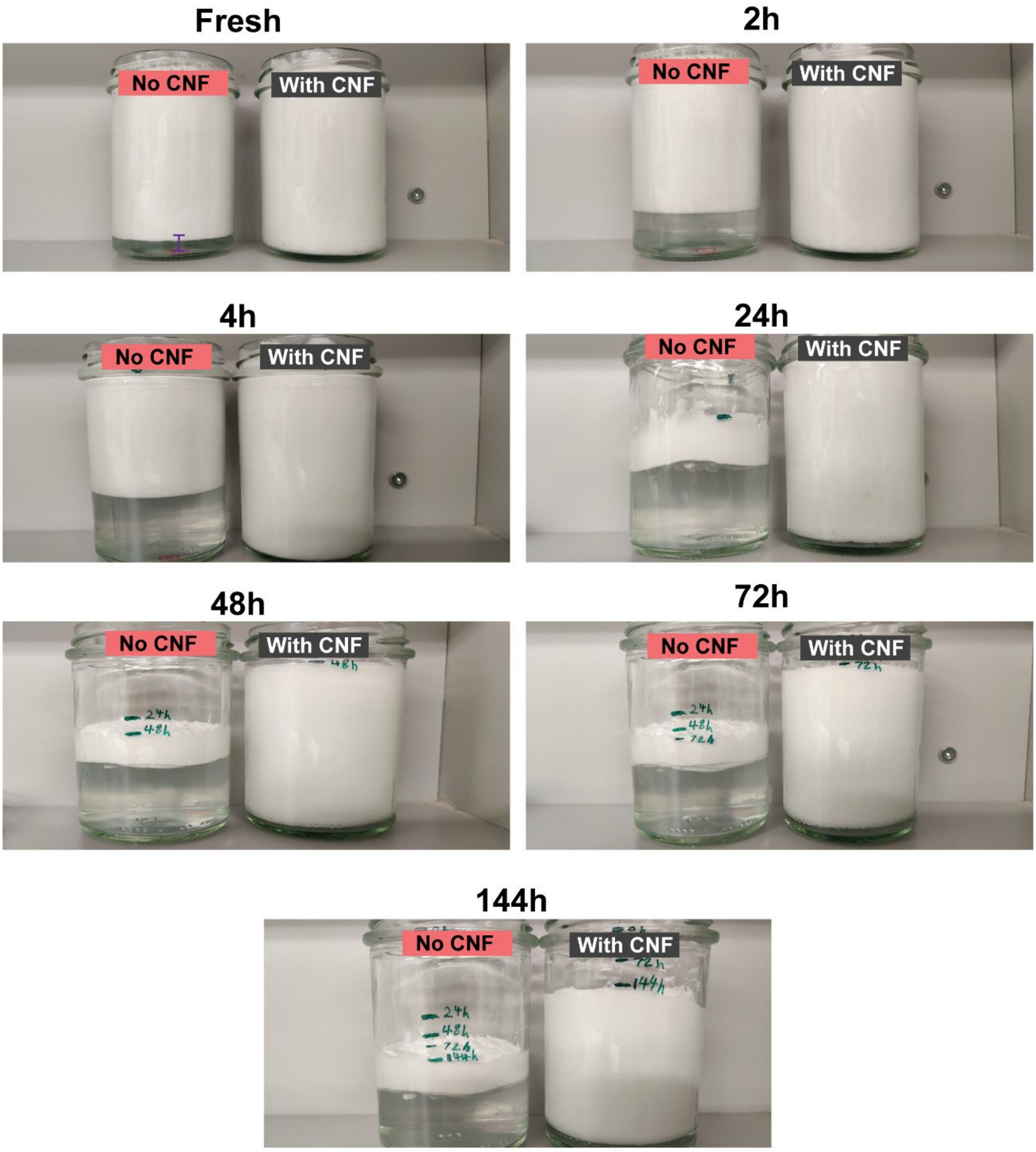

Figure S7. Stability of the Pickering foams in the air at 25% RH and 20 ºC over time.

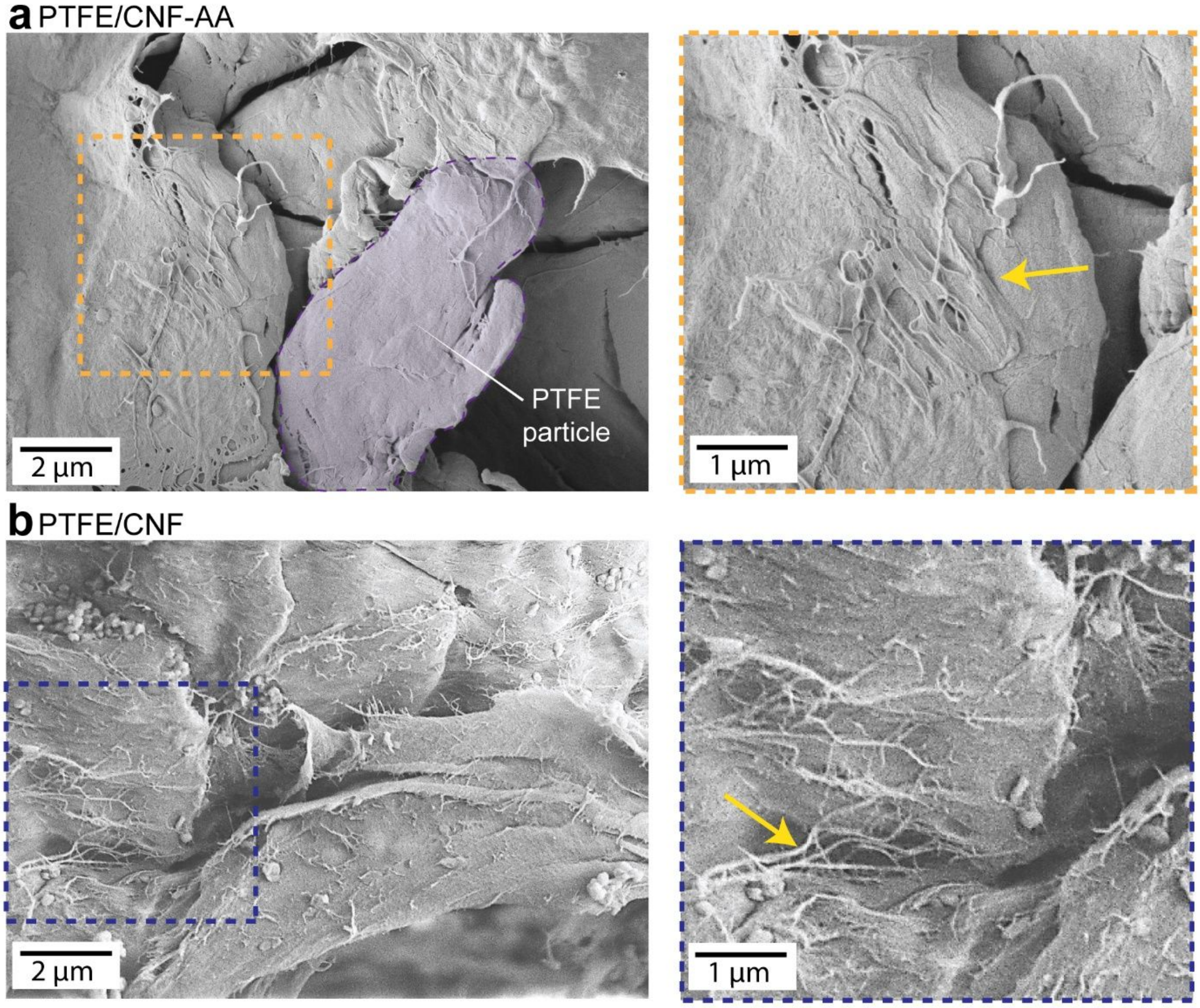

Figure S8. SEM images of supraparticles prepared with PTFE 35 µm bound by 15% of a) acetylated CNF and b) unmodified CNF. The panels on the right includes images of higher magnification.

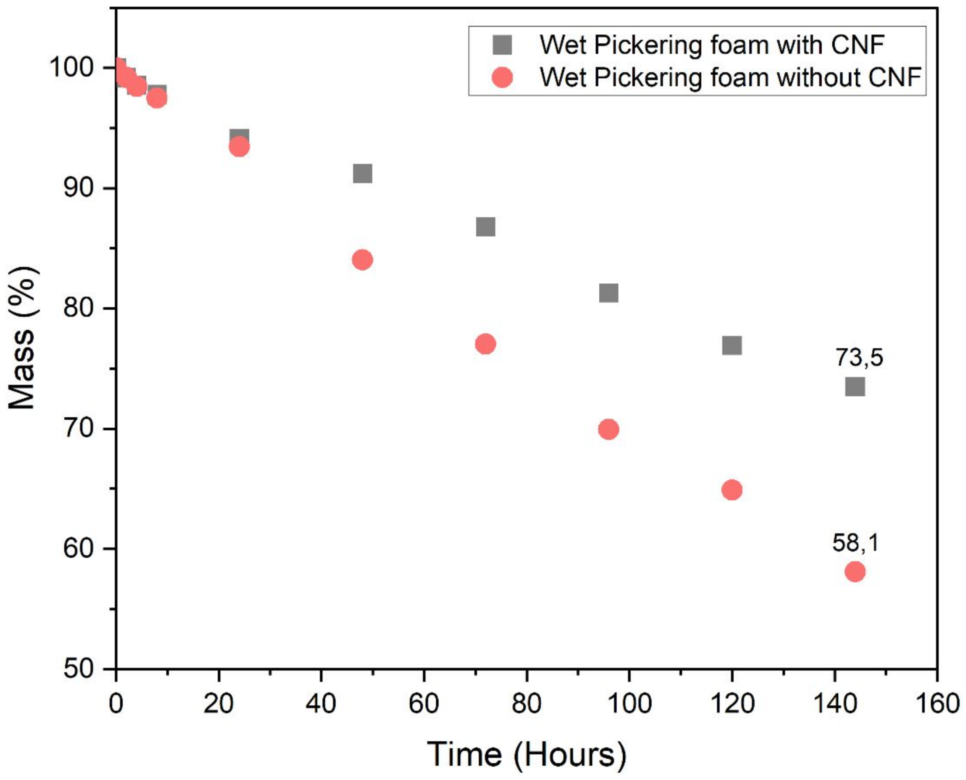

Figure S9. Kinetic mass loss over time comparison of wet Pickering foams with and without CNF as a function of time.

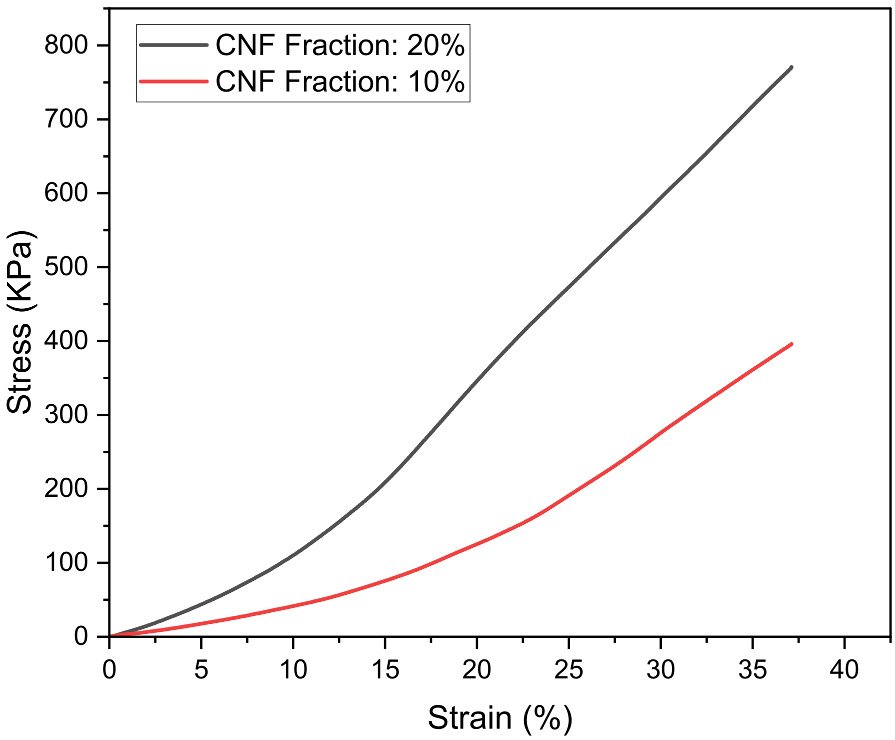

Figure S10. Stress-strain curves of dried Pickering foams with CNF fraction at 10 and 20% (cylindrical shape).
